# Identification and Functional Analysis of Long Non-Coding RNA (lncRNA) in Response to Seed Aging in Rice

**DOI:** 10.3390/plants11233223

**Published:** 2022-11-24

**Authors:** Yixin Zhang, Fan Fan, Qunjie Zhang, Yongjian Luo, Qinjian Liu, Jiadong Gao, Jun Liu, Guanghui Chen, Haiqing Zhang

**Affiliations:** 1 College of Agronomy, Hunan Agricultural University, Changsha 410128, China; 2 Guangdong Key Lab for Crop Germplasm Resources Preservation and Utilization/Agro-Biological Gene Research Center, Guangdong Academy of Agricultural Sciences, Guangzhou 510640, China

**Keywords:** lncRNA, seed aging, ssRNA-seq, rice

## Abstract

Many lncRNAs have been shown to play a vital role in aging processes. However, how lncRNAs regulate seed aging remains unknown. In this study, we performed whole transcriptome strand-specific RNA sequencing of samples from rice embryos, analyzed the differences in expression of rice seed lncRNAs before and after artificial aging treatment (AAT), and systematically screened 6002 rice lncRNAs. During the AAT period, the expression levels of most lncRNAs (454) were downregulated and only four were upregulated among the 458 differentially expressed lncRNAs (DELs). Cis- or trans-regulated target genes of the four upregulated lncRNAs were mainly related to base repair, while 454 downregulated lncRNAs were related to plant–pathogen interaction, plant hormones, energy metabolism, and secondary metabolism. The pathways of DEL target genes were similar with those of differentially expressed mRNAs (DEGs). A competing endogenous RNA (ceRNA) network composed of 34 lncRNAs, 24 microRNAs (miRNA), and 161 mRNAs was obtained. The cDNA sequence of lncRNA *LNC_037529* was obtained by rapid amplification of cDNA ends (RACE) cloning with a total length of 1325 bp, a conserved 5′ end, and a non-conserved 3′ end. Together, our findings indicate that genome-wide selection for lncRNA downregulation was an important mechanism for rice seed aging. LncRNAs can be used as markers of seed aging in rice. These findings provide a future path to decipher the underlying mechanism associated with lncRNAs in seed aging.

## 1. Introduction

Rice is one of the most important grain crops in China and the world. Rice seed vigor tends to decline under high temperatures and high humidity in south China, especially during storage and transportation, causing great losses to agricultural production. Due to the high cost of cryogenic cold storage technology and the inability to fundamentally solve the problem of seed vigor decline caused by seed aging and deterioration, the genetic characteristics of the seed aging process have become the focus of seed science research.

The rate of seed vigor decline during seed aging is mainly determined by genetic mechanisms, environmental factors and storage conditions also have an important effect on seed viability decline [1,2,3]. During storage and aging, the ability to maintain vitality is better in inbred rice seeds than in hybrid rice seeds. In addition, the rate of seed aging is largely affected by the environment, such as storage temperature, seed moisture content, and seed quality [4]. A number of studies on molecular markers related to seed vigor longevity have been performed. However, the number of quantitative trait loci (QTL) related to seed vigor maintenance and their location on chromosomes, genetic effects, and interactions with the environment have different results due to the different materials used, and the application of these molecular markers in marker-assisted selection needs further study [5,6,7]. In terms of cloning and functional studies of rice seed viability genes, acetaldehyde dehydrogenase 7 (*OsALDH7*) [8], the *cry1Ab/cry1Ac* gene, and its associated *Bacillus thuringiensis* (*Bt*) protein [9], HVAC 19 which homology with the acetyl CoA carboxylase (ACCase) gene [10], Lipoxygenase *3* (*LOX3*) [11], OsGRETCHENHAGEN3-2 (OsGH3-2) [12], and protein repair *L-isoaspartyl methyltransferase 1* (*OsPIMT1)* [13] are all associated with seed viability and longevity in rice. Gao et al. [14] identified more proteins related to seed longevity during natural aging, including redox regulation proteins (glutathione-related proteins and glyoxalase), DNA-damage-repair/toleration proteins, and late embryogenesis-abundant proteins, and hypothesized that seed viability retention capacity is the result of a combination of multiple factors. Chen et al. found that galactose and gluconic acid, which significantly negatively correlated with the germination percentage of seeds, can constitute potential metabolic markers of seed vigor and aging [15]. Studies have shown that secondary metabolites are related to seed vigor, for example tocopherol content is higher in high vigor seeds [16]. Resveratrol, dihydromyricetin [17], and dihydroquercetin (a flavonoid component) can improve seed vitality, and coumarin can effectively regulate seed germination [18]. In addition, abscisic acid (ABA) and gibberellin (GA) are key factors to regulate seed vigor [19,20].

Although numerous studies, including proteomics, metabolomics, genomics, transcriptomics, and miRNA expression differences have been conducted on the process of seed vigor deterioration, the regulatory mechanism of rice vigor decline remains unclear. In recent years, aging research has experienced unprecedented progress, revealing that the aging rate can be controlled to some extent through epigenetic pathways and biochemical processes [21]. With the rapid development of high-throughput sequencing technology, thousands of aging-related lncRNAs have been found. Mining the important functions of lncRNAs in regulating gene expression provides new ideas for completing the puzzle of seed aging mechanism.

LncRNAs are generally defined as RNA transcripts in eukaryotes that are more than 200 nucleotides in length and have no discernible protein-coding potential or extremely low coding capacity [22,23,24]. LncRNAs are capable of regulating gene expression at the epigenetic, transcriptional, and post-transcriptional levels, and are widely involved in biological growth and development, stress resistance, and physiological and pathological processes. LncRNAs are closely associated with aging, and an increasing number of aging-related lncRNAs have been identified in mammals. Some lncRNAs, such as *Xist* [25], *MALAT1* [26], *MEG3* [27], and *GAS5* [28] are downregulated in the aging process, indicating their function in suppressing senescence. The expression of *Xist* in aging cells decreases [25] and *MALAT1* in aging fibroblasts decreases [26]. Other lncRNAs, such as *HOTAIR* [29], *HEIH* [30], *TERRA* [31], and *H19* [32] are upregulated in senescent cells. 

There are relatively few studies on plant lncRNAs. Plant lncRNAs play key roles in flowering regulation [33], response to light [23], gene silencing [34], root organ formation [35], seedling photomorphogenesis [36], response to stress [37,38], and reproductive growth [24,39]. *COLDAIR* (COLD ASSISTED INTRONIC NONCODING RNA), which is only enriched in *FLC* (FLOWERING LOCUS C) chromatin, and the mutations that disrupt *COLDAIR* binding to polycomb repressive complex 2 *(PRC2*) lead to vernalization insensitivity [40]. Cold-induced long antisense intragenic RNA (*COOLAIR*) is physically associated with the *FLC* locus and accelerates transcriptional shutdown of *FLC* during cold exposure in *Arabidopsis* [41]. As decoys, lncRNAs are able to bind miRNAs and block the interaction between miRNAs and their specific target genes, known as endogenous target mimicry (eTM) [42]. lncRNA *IPS1* (INDUCED BY PHOSPHATE STARVATION 1) competitively binds to miR399, leading to the upregulation of *PHO2* (PHOSPHATE 2) [43].

The roles of rice lncRNAs have been experimentally and functionally characterized in a few cases, and these studies have confirmed that lncRNAs play a pivotal role in a number of important biological processes. For example, lncRNAs are involved in tolerance to biotic and abiotic stress, including disease resistance [44], heat stress [45], drought resistance [46], nitrogen starvation [47], cadmium stress [48], rice blast pathogen *Maganaporthe oryzae* resistance [49], and expression regulation of adjacent genes in rice [50,51]. LncRNAs play crucial roles in the regulation of growth and development in rice. LncRNA *TCONS_00023703* was highly expressed in developing seeds, and its mutant plants showed a significant decrease in grain length and 1000-grain weight [52]. Wang et al., suggested that expression-delayed lncRNAs in caryopses located on secondary branches (CSBs) may regulate the development of caryopses located on primary branches (CPBs) and CSBs [53]. *MISSEN*, a parent-of-origin lncRNA, is the first lncRNA identified as a regulator in endosperm development [54]. LncRNA *TL* (TWISTED LEAF) in rice maintains the flatness of rice leaves by regulating the expression of the *R2R3-MyB* gene [55]. Yang et al. [46] identified some lncRNAs, miRNAs, and mRNAs related to drought resistance in Shanlan upland rice. Fifty-six lncRNAs were discovered under arsenic stress and indicated that they may participate in signal transmission [56]. Liu et al. [57] revealed that lncRNAs participate in rice ovule development and female gametophyte abortion via various possible mechanisms. The single nucleotide polymorphism generated by the mutation changed the secondary structure of long-day-specific male fertility-associated RNA *(LDMAR*), which leads to enhanced methylation in the promoter region and reduced the transcription of *LDMAR* [58]. The DELs associated with transposable elements and meiosis-regulated targets might be endogenous non-coding regulators of pollen/embryo sac development that cause low fertility in autotetraploid rice [59]. Furthermore, in cereal crops and other crop species, changes in lncRNA expression may play an important role in the regulatory changes associated with crop domestication [60].

However, there are few studies on lncRNAs related to plant seed aging, especially in rice. In addition, it is necessary to study the interactions between lncRNAs and other ceRNAs to discover the regulation network. Here, lncRNA maps related to seed aging were analyzed to deepen the understanding of lncRNA expression and function and provide a new idea to complete the puzzle of the seed aging mechanism. LncRNA can be used as a candidate marker for seed aging, which provides an important basis for the improvement of plant varieties and seed vitality detection. The results indicate a bright direction for the successful utilization of lncRNAs in rice.

## 2. Results

### 2.1. Genome-Wide Identification and Characterization of LncRNAs in Rice

**Figure 1:**
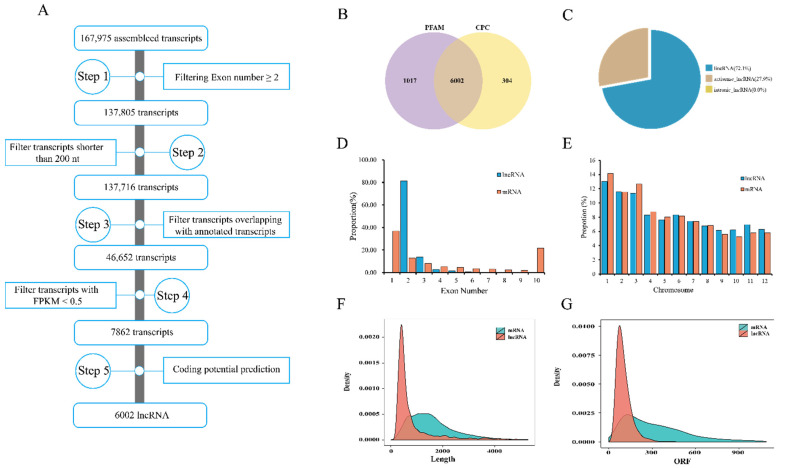
Identification and characterization of lncRNAs in rice. (**A**) Pipeline of lncRNA identification. Fragments per kilobase of exons per million fragments mapped, FPKM. (**B**) Venn diagram of lncRNAs filtered by the CPC [61] and PFAM [62] program. PFAM: a database of protein families; CPC: coding potential calculator. (**C**) Composition of multiple types of lncRNAs. Comparison of lncRNA and mRNA characteristics, including exon numbers (**D**), chromosome distribution (**E**), transcript length (**F**), and ORF length (**G**).

To investigate the role of lncRNAs in seed aging, strand-specific RNA sequencing (ssRNA-Seq) and bioinformatics were performed to analyze the differential changes in lncRNA expression before and after rice seed aging. Three biological replicates of aged seeds (50% germination percentage, S50) and new seeds (96% germination percentage, S96) were performed in this study. The six samples generated 84,333,888–119,102,852 clean reads (12.07–17.25 GB clean bases) with a Q30 of 89.20–93.93% and similar GC content, indicating that the quality of RNA-seq was highly reliable for subsequent analysis (Table S1). Of the clean reads, 68.21–76.63% were successfully aligned with the reference genome. After five rounds of filtering, we obtained 6002 lncRNAs (Figure 1A,B). Among the obtained lncRNAs, the number of intergenic lncRNAs (lincRNAs) was about twice as many as long non-coding natural antisense transcripts (lncNATs) (Figure 1C).

To further understand the characteristic differences in the sequence structure of lncRNAs and mRNAs identified in rice, we analyzed and compared lncRNAs and mRNAs according to the number of exons, chromosome distribution, sequence length, and open reading frame (ORF) length of transcripts. Most lncRNAs (more than 90%) had two or three exons, while the exon number of mRNAs was widely distributed (Figure 1D). Most lncRNAs were distributed on chromosomes 1, 2, and 3, and mRNAs and lncRNAs had similar distribution rates on these chromosomes (Figure 1E). Most sequence lengths of lncRNAs ranged from 200 to 1000 nt, while the length of mRNA sequences ranged from 800 to 3000 nt (Figure 1F). More than 90% of lncRNAs contained ORF length ≤200 nt, while 40% of mRNAs had an ORF length ≥200 nt. Overall, compared with mRNA, the transcript lengths and ORF lengths of the lncRNAs were shorter (Figure 1G).

**Figure 2:**
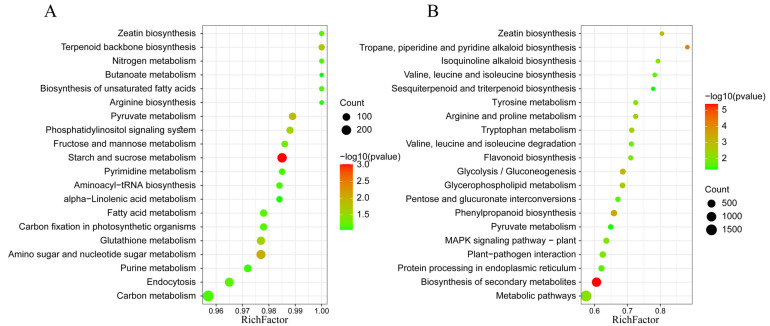
KEGG enrichment analysis of all detected lncRNA target genes. (**A**) KEGG pathway of cis-regulated target genes. (**B**) KEGG pathway of trans-regulated target genes. Rich factor refers to the ratio of the number of DEGs in the pathway and the gene number in the pathway of the total annotated genes in this species, and large rich factor indicates a high degree of enrichment. The area of each colored circle is proportional to the number of genes involved in each pathway; the color indicates the *p* value, and the *x*-axis is the rich factor.

Subsequently, we predicted the biological functions of all detected lncRNAs through cis- and trans-acting modes. In our study, the trans-regulated genes were predicted by co-expression analysis based on the expression level of lncRNAs among samples, and the proximal protein-coding genes located within a genomic window of 100 kb of lncRNAs (co-localization) were screened as their target genes for cis activity. Kyoto Encyclopedia of Genes and Genomes (KEGG) enrichment analysis of cis-regulated target genes showed lncRNAs involved in 138 KEGG pathways, which were mostly enriched in metabolic pathways (such as nitrogen metabolism, pyruvate metabolism, amino sugar and nucleotide sugar metabolism, and glutathione metabolism), energy metabolism (such as starch and sucrose metabolism), phosphatidylinositol signaling system, terpenoid backbone biosynthesis, and fructose and mannose metabolism. Pathways in the top 20 enrichment degrees are shown in Figure 2A. In the KEGG enrichment analysis of the trans-regulated target genes with lncRNAs, the target genes covered 138 KEGG pathways, among which phenylpropanoid biosynthesis, zeatin biosynthesis, plant–pathogen interaction, biosynthesis of secondary metabolites, MAPK signaling pathway plant, metabolic pathways, and flavonoid biosynthesis were significantly enriched (*p* < 0.05). Pathways in the top 20 enrichment degrees are shown in Figure 2B.

### 2.2. DELs and Functional Annotation during Artificial Aging of Rice Seeds

**Figure 3:**
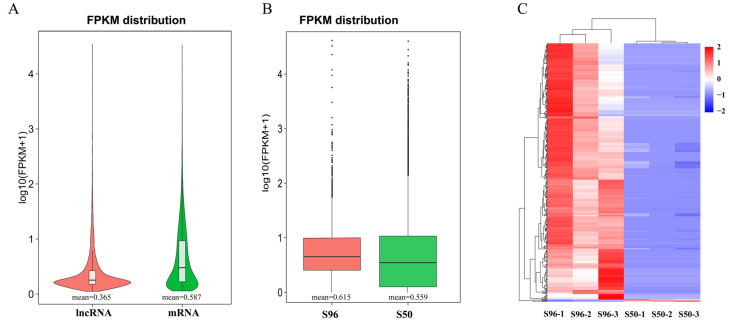
DELs during seed aging. (**A**) The distribution of the FPKM value of lncRNAs and mRNAs. (**B**) Comparison of lncRNA expression levels. (**C**) Heatmap of significant DELs in rice embryos. Each column in the heatmaps represents a sample, and each row represents a gene. Different colors indicate the expression of the gene in different samples, with darker red indicating higher gene expression and darker blue indicating lower gene expression.

By determining the expression of lncRNAs, we demonstrated that the average expression of these lncRNAs (0.365) was lower than that of mRNA (0.587) (Figure 3A). Compared with new seeds (S96, mean = 0.615), the average expression of lncRNAs in aged seeds (S50, mean = 0.559) was lower (Figure 3B). Most of the significant DELs were downregulated after seed aging treatment. Among them, 454 lncRNAs were significantly downregulated, and only 4 lncRNAs were upregulated (Figure 3C, Table S2). We validated randomly selected two downregulated lncRNAs, four upregulated lncRNAs and two downregulated mRNAs using quantitative real-time PCR (qPCR), and the results showed that the expression level was consistent with the sequencing results (Supplementary Figure S1).

The KEGG Orthology-Based Annotation System (KOBAS) was used to investigate the functions of the DEL cis- and trans-regulated mRNAs. According to the results of the KEGG classification, the target genes of significantly upregulated and downregulated lncRNAs were involved in different functional pathways. The only four upregulated lncRNAs were *LNC_001951*, *Os02t0591850-01*, *Os03t0332600-01*, and *Os01t0704250-00*. We predicted the functions of their possible cis- and trans-regulated genes and found that all four lncRNAs were in gene-dense regions, with many cis-regulated protein-coding genes both upstream and downstream. There were less trans-regulated genes, among which, *Os02g0591850* and *Os03g0332600* were highly correlated with the expression of many genes, while *LNC_001951* had only five genes whose Pearson correlation coefficient (PCC) was greater than 0.95, and *Os01g0704250* had no trans-regulated genes (Table S3).

According to the KEGG enrichment results, the corresponding cis-regulated target genes of the four upregulated lncRNAs were significantly enriched in homologous recombination and non-homologous end joining (Table S4). Among them, the target genes of *LNC_001951* were *Os04g0635900* (DNA repair exonuclease family protein) and *Os04g0637400* (similar to poli-like DNA polymerase). In addition, the cis-regulated target genes were involved in the mismatch repair and mRNA surveillance pathway, among them, the target gene of *Os02t0591850-01* is *Os02g0592300* (DNA mismatch repair protein). The target genes of lncRNA *Os01t0704250-00* were *Os01g0702900* (Similar to Sucrose-phosphate synthase) and *Os01g0703000* (SRP RNA 3′ adenylating enzyme) (Table 1). The four upregulated lncRNA trans-regulated target genes were significantly enriched in the mRNA surveillance pathway (Table S4), such as the target genes of *Os03t0332600-01* were *Os06g0319600* (Poly(A) polymerase), *Os04g0252200* (similar to CPSF160%3B nucleic acid binding), and *Os06g0563300* (Similar to serine/threonine protein phosphatase) (Table 1). Interestingly, 57.69% of trans-regulated KEGG pathways overlapped with cis-regulated KEGG pathways, and the overlapped KEEG pathways were associated with DNA repair and mRNA surveillance pathways, indicating that upregulation of target genes can maintain seed viability by regulating DNA damage repair and other pathways.

**Figure 4:**
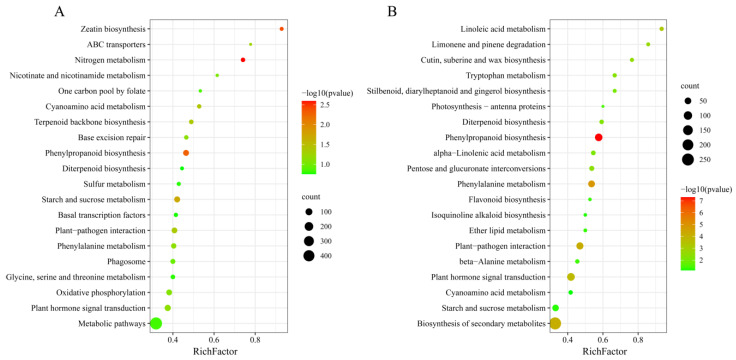
KEGG functional analysis of downregulated lncRNAs target genes. (**A**) KEGG pathway of downregulated cis-regulated target genes. (**B**) KEGG pathway of downregulated trans-regulated target genes.

There were 454 downregulated lncRNAs whose Gene Ontology (GO) (Table S5) and KEGG functional enrichment analyses of trans-regulated and cis-regulated target genes were extensive. The top ten KEGG enrichment pathways of cis-regulated genes with downregulated lncRNAs included nitrogen metabolism, zeatin biosynthesis, phenylpropanoid biosynthesis, starch and sucrose metabolism, cyanoamino acid metabolism, plant–pathogen interaction, terpenoid backbone biosynthesis, ABC transporters, plant-hormone signal transduction, and phenylalanine metabolism (Figure 4A). Among them, plant–pathogen interaction, plant-hormone signal transduction, and secondary metabolism-related phenylpropanoid biosynthesis and phenylalanine metabolism pathways were also included in the top ten enrichment pathways of trans-regulated genes. Starch and sucrose metabolism were also enriched in the trans-regulated genes (Figure 4B).

It is worth noting that all trans-regulated target genes are significantly enriched in biosynthetic pathways related to plant secondary metabolites, such as Os02g0767300 (flavonol synthase), Os10g0320100 (flavonoid 3′-monooxygenase), Os03g0367101 (flavonoid 3′,5′-hydroxylase), Os02g0701600 (tocopherol O-methyltransferase), Os12g0190000 (GDP-L-galactose phosphorylase, key enzymes for vitamin C synthesis), Os01g0508000, Os07g0656200, and Os01g0771900 (beta-glucosidase, key enzymes regulating cumarin synthesis), Os06g0626700 (anthocyanidin synthase), and hormone-related enzyme Os05g0178100 (gibberellin 3beta-dioxygenase), Os04g0517600 (gibberellin 3beta-dioxygenase), and Os07g0281700 (abscisic-aldehyde oxidase)) (Table S6).

### 2.3. DEGs and Functional Annotation during the Artificial Aging of Rice Seeds

**Figure 5:**
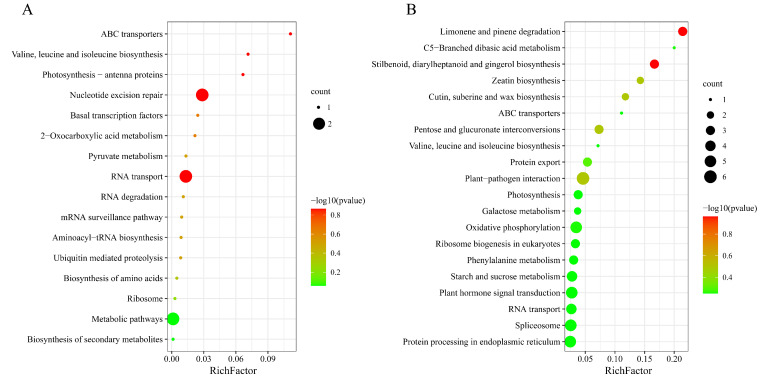
KEGG classification of significant DEGs. (**A**) KEGG pathway of significantly upregulated mRNAs. (**B**) KEGG pathway of significantly downregulated mRNAs.

Sequencing results of new seeds and aged seed embryos showed that mRNA expression was also predominantly downregulated, with 55 significantly upregulated and 1171 significantly downregulated DEGs. In addition, the KEGG classification of significantly downregulated DEGs differed from that of upregulated DEGs. The upregulated genes were classified into three main pathways: the nucleotide excision repair (NER) pathway, pathways associated with transcription and translation, and metabolic pathways (Figure 5A). The KEGG classification of downregulated genes was mainly enriched in plant–pathogen interaction, plant hormone-related pathways (plant hormone signal transduction, zeatin biosynthesis), energy metabolism and biosynthesis of other secondary metabolites, transcription and translation, and protein (folding, sorting, and degradation) (Figure 5B).

Sequencing results showed that the pathway of DEL trans-regulated and cis-regulated target genes were similar with the results of DEGs, suggesting that the seeds underwent a decline in energy metabolism, damage, degradation, and repair of DNA, RNA, and protein in the process of deterioration and near death, and many lncRNAs and related genes involved in the process of stress resistance and base repair.

### 2.4. Potential lncRNA-miRNA-mRNA ceRNA Network in Rice Seed Aging

**Figure 6:**
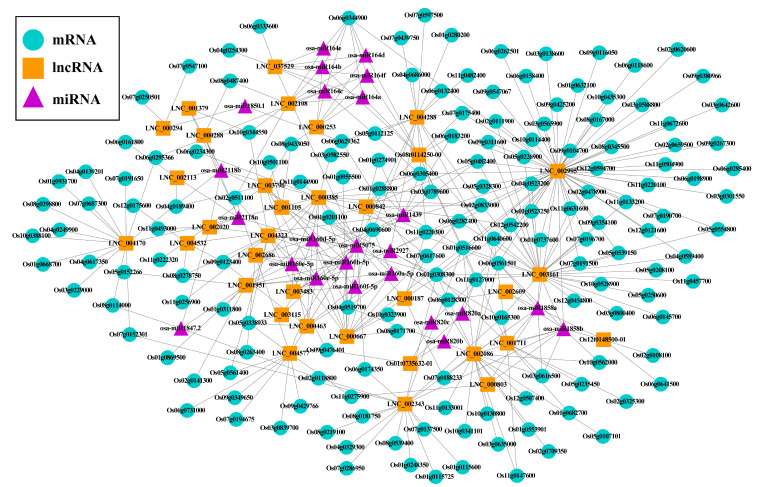
CeRNA network for predicting the interaction between lncRNAs, miRNAs, and mRNAs during rice seed senescence.

One of the most important functions of lncRNAs is to act as ceRNAs, which can competitively bind miRNA, relieve its inhibition of target genes, and form a complex ceRNA regulatory network. Therefore, a ceRNA network was constructed to predict the interaction between lncRNAs, miRNAs, and mRNAs in the aging process of rice seeds. In this study, significant DELs and DEGs of the lncRNA–mRNA trans-regulated network (*p* > 0.95) were used as the prediction library of miRNAs. A total of 713 mature miRNAs were downloaded to predict lncRNAs and target mRNAs. First, 314 miRNAs and 318 lncRNAs were predicted to constitute 1847 relationship pairs using RNAhybrid software (Table S7), and 110 miRNAs targeted 73 mRNAs, forming 178 miRNA–mRNA relationship networks, as shown using the online tool psRNATarget (Table S8). Finally, the miRNA–lncRNA and miRNA–mRNA networks with the same miRNA were merged. The ceRNA network comprised 34 lncRNAs, 24 miRNAs, and 161 mRNAs (Figure 6, Table S9). The identified miRNAs included *osa-miR160a-5p*, *osa-miR164a*, and *osa-miR820b*, among which the *osa-miR160* and *osa-miR164* families were the largest, with 6 miRNA members each.

To explore the role of mRNAs in the ceRNA network, mRNA was annotated with GO function, and 90 mRNAs were annotated into 761 GO terms (Tables S10 and S11). Among them, the most enriched class was biological process, followed by 206 molecular functions, and 117 GO terms were significantly enriched (*p* value < 0.05). The genes were significantly enriched in the biological process GO term, including GO:0007154 cell communication, GO:0009755 hormone-mediated signaling pathway, GO:0032870 cellular response to hormone stimulus, GO:0007165 signal transduction. KEGG pathway analysis of mRNAs participation in the ceRNA network showed that 33 of the 161 mRNAs involved in 17 pathways, 4 of which were significantly enriched such as glyoxylate and dicarboxylate metabolism, glycine, serine, and threonine metabolism (Table S12).

### 2.5. Cloning of Full-Length cDNA of Seed Aging-Related lncRNA LNC_037529

**Figure 7:**
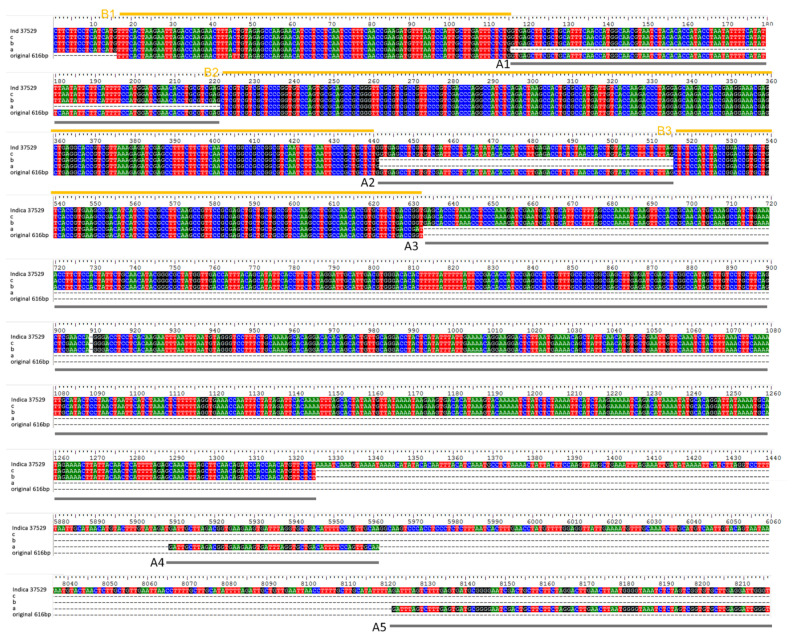
Alignment of the *LNC-037529* sequence with the indica rice genome. A1 to A5 were the exons which can be span in alternative splicing, B1 to B3 were common sequences of all alternative splicing.

Since *LNC_037529* showed lower levels in aged seeds than in new seeds using more rice varieties in the qPCR verification experiment (Supplementary Figure S2), we cloned *LNC_037529* as an aging-related lncRNA to further investigate and verify its function in the seed aging process and obtained a full-length cDNA sequence. A comparison with the indica genome showed that lncRNA *LNC_037529* had different alternative splicing (Figure 7). A1 and A2 were two intron splicing sites with GT-AG. The 5′ end of lncRNA *LNC_037529* was very conserved, and the conserved sequence length was 633 bp. The 3′ end was not conserved, and the transcript was very long. The first transcript was about 1325 bp, the second was 8216 bp, and the last two exons spanned 4584 bp and 2160 bp introns. In sequence A, 188–335, 450–506, and 506–766 bp were transposon origin sequences, and a large segment of LTR retrotransposon insertion occurred in this location in japonica and indica rice.

## 3. Discussion

### 3.1. A Large Amount of lncRNA Was Degraded during Seed Aging

In this study, 6002 rice candidate lncRNAs were identified. The number of lncRNAs was about twice as many as lncNATs, and the proportion was similar to the results reported by Zhang [63]. This group of lncRNAs may be informative for functional genomic studies in rice, especially regarding the mechanisms associated with seed aging. LncRNAs are highly species-specific, tissue-specific, and developmental stage-associated [64]. When comparing samples from different aging states, the expression of the vast majority of lncRNAs was found to be downregulated in seeds after aging, and only 4 lncRNAs were upregulated. In the study of Yang et al. [46], a total of 91 DELs were obtained in upland rice under drought stress, including 141 upregulated and 50 downregulated lncRNAs, which was different from our results. Xu et al. obtained 78 significantly differentially expressed lncRNAs, including 67 upregulated lncRNAs and 11 downregulated lncRNAs in the *Populus x canadensis moench* heat stress response experiment [65]. In addition, 26 differentially expressed lncRNAs were obtained in *Populus qiongdaoensis* seedlings under heat stress conditions, among which 25 were upregulated and one was downregulated, in contrast to our results [66]. In our study, the vast majority of lncRNAs decreased during seed aging, suggesting that lncRNAs have special functions during seed aging and that lncRNAs can be used as markers of aging seeds. Meanwhile, the percentage of downregulated lncRNAs was significantly higher than that of mRNAs, suggesting that lncRNAs performing regulatory and structural functions are more sensitive than mRNAs during aging.

### 3.2. Possible Mode of Action of lncRNA

LncRNA can regulate target genes in cis- and trans-ways, and the corresponding analysis methods of co-localization and co-expression can be used to predict the function of lncRNAs [40,41,46,63]. Many studies have been reported based on the cis-regulated target genes of lncRNAs. The most well-known lncRNAs in plants, such as *COOLAIR* and *COLDAIR*, are closely linked to the expression and regulation of their cis-regulated genes [40,41]. It was found that chromatin-enriched lncRNAs may be local enhancers that affect the expression of multiple genes in rice [63]. In addition to cis-regulated relationships, trans-regulated networks can be considered in the analysis process. Based on the known functions of trans-regulated mRNAs, the function and potential regulators of candidate lncRNAs can be predicted. Increasingly, trans-acting lncRNAs also regulate site-independent gene expression [44,46].

We predicted the functions of target genes in the cis and trans-acting modes of the detected lncRNAs. Based on the results of the KEGG classification of cis-regulated and trans-regulated genes of lncRNAs, the number and functional pathways of DELs that were significantly up- and downregulated in aging seeds differed. Only 4 lncRNAs were upregulated, and the function of their trans- and cis-regulated target genes were related to base repair, among other functions. While 454 lncRNAs were downregulated, the pathways of their trans- and cis-regulated target genes were related to plant–pathogen interactions, plant hormone, energy metabolism, and secondary metabolism, which was similar to DEGs before and after AAT.

It is interesting that all trans-regulated target genes are significantly enriched in biosynthetic pathways related to plant secondary metabolites. Secondary metabolites are the result of the adaptation of plants to the ecological environment in the long-term evolution, and play important roles in dealing with the relationship between plants and ecological environment [67]. Among the secondary metabolites, plant flavonoids are crucial in mediating plant responses to biological and abiotic environmental factors due to their antioxidant activities [68]. Flavonoids, such as curcumin and quercetin, play a crucial role in plant defense, anti-cancer, and regulating the expression of lncRNA [68,69]. Similar to flavonoids, which eliminate reactive oxygen species (ROS) and activate antioxidant enzymes to prevent damage caused by free radicals, tocopherol (vitamin E) and vitamin C also participated in the defense reaction of lipid oxidation by regulating gene expression, extending seed life and protecting seedling lipid oxidation [70,71]. In addition, hormones such as ABA and GA are also key factors in the regulation of seed vigor, and GA and vitamin E treatment have the effect of improving seed vigor and promoting seed germination [20]. In this study, the trans-target genes of all detected lncRNAs (Figure 2B) in seed embryos and DELs (Figure 4B and Figure S3) were significantly enriched in secondary metabolic pathways, especially the flavonoid biosynthesis pathway, phenylpropanoid biosynthesis, and plant hormone signal transduction pathway. Related genes included gibberellin 3beta-dioxygenase, abscisic-aldehyde oxidase, flavonol synthase, tocopherol O-methyltransferase, peroxidase, etc. The results indicate that lncRNA played an important role in regulating secondary metabolites to improve the self-protection and survival competitiveness of seeds [67]. This finding provides a theoretical basis for the use of secondary metabolites to regulate seed vigor.

It is generally accepted that the decline of seed vigor during aging is related to changes in cellular, metabolic, and biochemical levels, including loss of membrane integrity, decreased energy metabolism, impaired RNA and protein synthesis, and DNA degradation [2]. Moreover, free radical scavenging systems, redox-regulated proteins, as well as DNA-damage-repair/toleration proteins are associated with seed storage tolerance during seed aging deterioration [14,72].

At high temperature, resistance-related functions shut down, leaving only survival-related basic functions [73]. Under certain conditions, plants may prioritize resources and components that can be shared. Defense responses at higher temperatures can be strengthened by lowering hormone levels [73]. In this study, both DEGs and lncRNA target genes showed a significant decrease in the expression of plant pathogen-related genes, secondary metabolism-related genes, and hormone-related genes, while the expression of base repair-related genes increased significantly in aging seeds. It is implied that in the process of aging deterioration, seeds experience a decline in stress tolerance and energy metabolism, damage, degradation, and repair of DNA, RNA, and protein, the gradual depletion of antioxidant systems and secondary metabolites (e.g., flavonoids, vitamin C, vitamin E and hormones). Most lncRNAs and mRNAs are degraded in the process of aging and deterioration to near death. However, 4 lncRNAs were upregulated, which involved base repair, suggesting they could be playing an import role in maintaining seed viability during seed aging.

### 3.3. Potential lncRNA–miRNA–mRNA ceRNA Network in Rice Seed Aging

Increasing evidence emphasizes the important role of lncRNAs as ceRNAs in biological processes [74]. The mutual regulation of miRNAs, lncRNAs, and mRNAs in plants may play a crucial role in the way plants resist or tolerate high temperature stress [66]. Studies have shown that lncRNA not only directly regulates mRNA but also affects the expression level of its target genes by controlling the expression of miRNA. LncRNAs carry some “seed sequence” of miRNA, which binds miRNA, thus preventing miRNA from binding to its target mRNA. LncRNAs can act as ceRNAs or miRNA adsorbers to regulate miRNAs [74]. lncRNA *IPS1* induced by phosphate starvation was identified, which binds *ATH-mir399* to form trinucleotide spikes between positions 10 and 11 at the 5′ end. *IPS1* functions as a target mimicry of *miR399* and inhibits *miR399*-mediated *PHO2* site-specific cleavage [75]. The pairing abolished the cleavage effect of *mir-399* on *IPS1*. Therefore, *IPS1* can be used as a decoy to induce *miR-399* to interfere with *ath-miR399* binding to other targets, which is a functional endogenous *miR399* target mimicry (eTM).

In our study, computational methods were used to construct a ceRNA network, which included 34 lncRNAs, 24 miRNAs, and 161 mRNAs. Among these, 33 lncRNAs and 160 mRNA were downregulated, but only one lncRNA (*LNC_001951*) and one mRNA (*Os06g0128300*) were upregulated. The largest of them were the *osa-miR160* and *osa-miR164* families, each with 6 miRNA members. Qin et al. [76] demonstrated that the expression of *miR164c* decreased accordingly when the miRNA related to rice seed viability decreased until it lost its viability. However, the expression of *miR164c* increased significantly in the inactive seeds. *MiR164c*-related lncRNA (*Lnc_000253*) and its mRNA were downregulated, which strongly suggests that lncRNA and *miRNA164c* play a reverse regulatory role in response to seed aging. GO and KEGG enrichment analyses were performed for mRNA in the lncRNA-miRNA-mRNA network. The most highly enriched GO terms were “GO:0007154 Cell Communication”, “GO:0009755 hormone-mediated Signaling Pathway”, “GO:0032870 Cellular Response to Stimulus” and “GO:0007165 Signal Transduction”. From the KEGG results, we found that mRNA was significantly enriched in the glyoxylate and dicarboxylate metabolism and glycine, serine, and threonine metabolism pathways, and gene annotation confirmed that most genes were proteins with unknown function. These unknown proteins hinder our understanding of the function of target genes in the ceRNA network. In future research, our focus will be on revealing the function of these unknown proteins in the ceRNA network.

## 4. Materials and Methods

### 4.1. AAT and Germination Experiment

Experimental rice (*Oryza sativa* L.) seed material (Rice variety R998) was obtained from the Rice Research Institute of Guangdong Academy of Agricultural Sciences. Rice variety R998 was planted in Guangdong Academy of Agricultural Sciences, Guangzhou, Guangdong Province (N: 113.273, E: 23.1579, PH6.5) with 4 experimental plots of 66.7 m^2^ each. Half of the freshly dried seeds harvested were used for the aging experiment and half for the controls. Mature seeds were harvested and dried, then stored at 4 °C for 30 days before AAT. AAT was conducted following Chen et al. [77] and Liu et al. [78] with minor modifications. The specific method of AAT treatment in this study was as follows: Each step was performed in an airtight container containing an appropriate saturated solution of salts to obtain stable relative humidity (RH). Three sealed containers were placed in dark at the different temperatures for various numbers of days. Temperature and RH were monitored with controllers placed inside the containers. First, 100 g seeds of four biological replicates were placed in mesh bags and equilibrated for 3 days at 85% RH (15 °C) in the appropriate saturated solution of KCl. Then, they were transferred to the equilibrated container with an appropriate saturated solution of KCl (43 °C and 85% RH) for 8 d. Subsequently, seeds were dried for 3 days at 32% RH (25 °C) in the sealed containers with appropriate saturated solution of MgCl_2_. The germination percentage of seeds with AAT (aged seeds) was 50% (S50). Seeds without AAT (new seeds) were used as controls, which germination percentage was 96% (S96).

Two layers of filter paper were laid in a 10 × 10 cm germination box, and AAT seeds and control seeds were laid flat in the germination box with 20 mL distilled water. Four groups of 100 seeds each were taken for each material, and the germination percentage was counted after 7 days. Germination occurs when the embryo breaks through the seed coat by 2 mm.

### 4.2. Extraction of Total RNA from Embryos of Rice Seed

Total RNA of rice seed embryos (the picture of seed embryo was shown in Supplementary Figure S4) was extracted according to an RNA extraction kit (Plant/Fungi Total RNA Purification Kit, NorgenBiotek Corp, Thorold, ON, Canada) and a genome removal kit (Rnase-free DNase I Kit, NorgenBiotek Corp) instructions with three biological replicates.

### 4.3. RNA Quantification and Qualification

RNA degradation and contamination were monitored on a 1% agarose gel. RNA purity was checked using a NanoPhotometer^®^ spectrophotometer (IMPLEN, Westlake Village, CA, USA). The RNA concentration was measured using the Qubit^®^ RNA Assay Kit with a Qubit^®^ 2.0 Flurometer (Life Technologies, Carlsbad, CA, USA). RNA integrity was assessed using the RNA Nano 6000 Assay Kit of the Bioanalyzer 2100 system (Agilent Technologies, Santa Clara, CA, USA).

### 4.4. Library Preparation for lncRNA Sequencing

A total of 3 μg RNA per sample was used as input material for the RNA sample preparations. First, ribosomal RNA was removed using the EpicentreRibo-zero™ rRNA Removal Kit (Epicentre, Madison, WI, USA), and the rRNA-free residue was cleaned up by ethanol precipitation. Subsequently, sequencing libraries were generated using the rRNA-depleted RNAbyNEBNext^®^ Ultra™ Directional RNA Library Prep Kit for Illumina^®^ (NEB, Ipswich, MA, USA) following the manufacturer’s recommendations. Briefly, fragmentation was carried out using divalent cations under elevated temperature in NEBNext First Strand Synthesis Reaction Buffer (5X). First-strand cDNA was synthesized using a random hexamer primer and M-MuLV Reverse Transcriptase (RNaseH-). Second-strand cDNA synthesis was subsequently performed using DNA Polymerase I and RNase H. In the reaction buffer, dNTPs with dTTP were replaced with dUTP. The remaining overhangs were converted into blunt ends via exonuclease/polymerase activities. After adenylation of 3′ ends of DNA fragments, NEBNext Adaptor with hairpin loop structure was ligated to prepare for hybridization. To select cDNA fragments that were preferentially 150–200 bp in length, the library fragments were purified with the AMPure XP system (Beckman Coulter, Beverly, MA, USA). Then, 3 μL USER Enzyme (NEB) was incubated with size-selected, adaptor-ligated cDNA at 37 °C for 15 min, followed by 5 min at 95 °C before PCR. PCR was then performed with Phusion High-Fidelity DNA polymerase, Universal PCR primers and Index (X) Primer. Finally, the products were purified (AMPureXPsystem, Beckman Coulter), and the library quality was assessed using the Agilent Bioanalyzer 2100 system. All primers used for RACE, nested PCR, and PCR are presented in Table S13.

### 4.5. Clustering and Sequencing

Clustering of the index-coded samples was performed on a cBot Cluster Generation System using the TruSeq PE Cluster Kit v3-cBot-HS (Illumia), according to the manufacturer’s instructions. After cluster generation, the libraries were sequenced on an Illumina Hiseq 2000 platform, and 100 bp paired-end reads were generated.

### 4.6. 5′ and 3′ RACE

Total RNA was extracted using a test kit. Using diluted 3′-cDNA as a template, PCR amplification was performed using primer GSP and reverse connector primer UPM in 3′ RACE primers. PCR products were diluted to 3′-cDNA1 as a template, and the second round of nest PCR was carried out with the combination of positive specific primer NGSP and reverse joint type primer UPMS in 3′ RACE primer. After the reaction of two rounds of PCR was completed, the reaction products were detected by 1.2% agarose gel electrophoresis, the target bands were recovered by gel cutting, ligated to the cloning vector, transformed into *Escherichia coli* (*E. coli*), and positive clones were screened and sequenced. Diluted 5′-cDNA was used as a template for the first round of PCR with GSP in the 5′ RACE primer and reverse splice primer UPM. The sequence of the 5′ end of the target gene was amplified using the 5′-RACE technique. The PCR product was diluted to 5′-cDNA1, and the second round (nested) PCR was performed with 5′-cDNA1 as the template, forward specific primer NGSP, and reverse splice sugary primer UPMS in 5′ RACE with the combination. After two rounds of PCR were completed, the reaction products were detected using 1.2% agarose gel electrophoresis, and the target bands were recovered by gel cutting, ligated to cloning vectors, transformed into *E. coli*, screened, and sequenced for positive clones [79].

### 4.7. Transcriptome Data Processing

Clean data (clean reads) were obtained by removing reads containing an adapter, reads containing ploy-N, and low-quality reads from raw data using Trimmomatic [80] in fastq format. The Q20, Q30, and GC content of the clean data were calculated using in-house perl scripts. All downstream analyses were based on clean, high-quality data. The coding potential calculator (CPC) program was used to evaluate the coding potential of the transcripts [61].

The genomic sequences and annotation of *O. sativa* ssp. *Japonica*. Cv. ‘Nipponbare’ was downloaded from the website (https://plants.ensembl.org/ accessed on 5 January 2022). The index of the reference genome was built using Bowtie (v2.0.6) [81], and paired-end clean reads were aligned to the reference genome using Tophat (v2.0.9) [82]. The expression level of each transcriptome was measured using the FPKM method calculated by Cufflinks (v2.2.1) with default parameters [83]. DEGs and DELs were analyzed using the R package DESeq2 and identified as DEGs with the criteria of |log2FoldChange| > 1 and *p*-value < 0.05. A heatmap and Venn diagrams of gene expression were generated by TBtools software [84].

### 4.8. Transcriptome Assembly and lncRNA Identification

Before screening, Cuffmerge software was used to merge the transcripts obtained by splicing each sample, and the transcripts with an uncertain chain direction were removed to obtain complete transcriptome information for this sequencing. Subsequently, lncRNA identification was performed on the merged transcript sets based on the following steps. (1) A large number of single exon transcripts with low expression and low confidence were screened from the splicing results of the transcriptome, and transcripts with an exon count ≥2 were selected. (2) Transcripts with a length ≥200 bp were selected. (3) Cuffcompare software was used to screen out transcripts that overlapped with exon regions of database annotations, and lncRNAs that overlapped with exon regions of this spliced transcript in the database were included in subsequent analysis as database annotated lncRNAs. (4) Cuffquant was used to quantify the expression level of each transcript, and transcripts with an FPKM ≥0.5 were selected. (5) Coding potential was the key condition for determining whether the transcript was lncRNA [85].

### 4.9. Prediction and Functional Enrichment Analysis

The lncRNAs were predicted to function by regulating the expression of prospective target genes in a cis- or trans-acting manner. LncRNA cis-regulated target genes are mainly predicted based on the positional relationship between lncRNAs and target genes. The protein-coding genes were screened as cis-regulated target genes (co-localized genes) within 100 kb upstream or downstream of the lncRNAs using Perl script [86]. Target genes were predicted to act in trans on the related lncRNA genes using the Pearson correlation coefficient (PCC) method. The lncRNA–mRNA pairs were considered to be trans-regulated when the PCC between lncRNAs and mRNAs was greater than 0.95 and the *p* value was less than 0.01 [86].

Subsequently, the potential functions of DELs and their cis- and trans-regulated genes were analyzed by Gene Ontology (GO) using the GOseq R package [87] and Kyoto Encyclopedia of Genes and Genomes (KEGG) [88] pathway enrichment analysis (KOBAS 3.0) [89]. The GO terms with a *p* value < 0.05 were defined as significantly enriched.

### 4.10. Validation by Quantitative Real-Time PCR

The first-strand cDNA was synthesized according to the instructions of iScriptTM cDNA Synthesis Kit (BIO-RAD, Hercules, CA, USA). The synthetic cDNA was then quantitatively analyzed in a CFX96™ Real-Time System (C1000™ Thermal Cycler, Bio-Rad). Each reaction was performed three times. After the experiment, the amplification curve and dissolution curve obtained in the quantitative real-time PCR (qPCR) experiment were analyzed. Using *UBQ5* as the internal reference gene, the obtained results were analyzed in Bio-Rad CFX manager 3.1 software of the qPCR system, and GraphPad Prism 9 software was used to process and analyze the data and calculate the expression difference of the target gene according to the 2^–ΔΔCT^ algorithm. All primers used are presented in Table S12.

### 4.11. CeRNA Network Construction

Rice mature miRNAs were downloaded from the miRbase database [90], and the RNAhybrid program [91] was used to predict miRNA–lncRNA trapping targets. Target mRNAs of miRNAs were predicted using the psRNATarget program [92]. The parameters used by the software refer to Song et al. [45]. Finally, the lncRNA and mRNA pairs sharing the same miRNA were selected to construct the competing endogenous RNAs (ceRNA) network. Cytoscape 3.7.2 [93] was used to display the regulatory network between lncRNAs, miRNAs, and mRNA.

## 5. Conclusions

In this study, more than 6000 lncRNAs were identified from the aging process of rice seeds. During the AAT period, the expression levels of most lncRNAs (454) were downregulated, and only four were upregulated. The functional analysis of cis- and trans-regulated target genes of DELs showed that the four upregulated lncRNAs were mainly involved in base repair to maintain seed viability, while the 454 downregulated lncRNAs were related to plant–pathogen interaction, plant hormones, energy metabolism, and secondary metabolism in the process of aging and deterioration to near death. The pathways of the DEL target genes were similar with those of DEGs. In addition, we constructed a ceRNA network composed of 34 lncRNAs, 24 microRNAs (miRNA), and 161 mRNAs and speculated that the ceRNA regulatory network played an important role in the seed aging process. The cDNA sequence of lncRNA *LNC_037529* was obtained by RACE cloning with a total length of 1325 bp, a conserved 5’ end, and a non-conserved 3′ end. Three sequence fragments were all transposons, and a large segment of LTR retrotransposons was inserted into rice. Together, our findings indicate that seeds undergo a decline in energy metabolism, damage, degradation, and repair of DNA, RNA, and protein in the process of aging and deterioration to near death. Most lncRNAs and mRNAs are degraded in the process of aging. However, four lncRNAs were upregulated, which involved in base repair to maintain seed viability during seed aging. The findings may provide a future path to decipher the underlying mechanism associated with lncRNAs in seed aging.

## Figures and Tables

**Table 1:** Description of corresponding cis- and trans-regulated target genes of upregulated lncRNAs.

Number	LncRNA_id	S50 FPKM	S96 FPKM	Status	Target Prediction Method	Target Gene ID	Target_Description
1	LNC_001951	35,512.70	11,679.20	Novel lncRNA	cis-regulation	Os04g0635900 Os04g0637400 Os04g0636100 Os04g0636900	DNA repair exonuclease family protein Similar to PolI-like DNA polymerase Glycosyl transferase, family 8 protein RNA-binding region RNP-1
					trans-regulation	Os09g0347700 Os02g0141300	Similar to Sec61p Mevalonate/galactokinase
2	Os02g0591850-01	24.79	8.94	Annotated lncRNA	cis-regulation	Os02g0593100| Os02g0592200| Os02g0592300| Os02g0591700	Similar to OSIGBa0106G07.12 protein Similar to alkaline phosphatase D DNA mismatch repair protein DNA mismatch repair protein
					trans-regulation	Os09g0536000 Os04g0592700 Os02g0139500 Os03g0349000 Os12g0176500 Os10g0487300 Os04g0473400 Os05g0565050 Os05g0182800	Exodeoxyribonuclease III xth family protein WD40 repeat-like domain containing protein Similar to Cycloartenol synthase Nucleoporin interacting component family protein Similar to Replication factor C subunit RFC4 Forkhead-associated domain containing protein Similar to 60S ribosomal protein Hypothetical conserved gene Similar to glutaminyl-tRNA synthetase
3	Os03t0332600-01	46.80	16.45	Annotated lncRNA	cis-regulation	Os03g0332400 Os03g0333400 Os03g0332500 Os03g0331700 Os03g0333300	Glyoxalase II Similar to photosystem II 11 kD protein Ribosomal protein L10e (IPR001197) Similar to cDNA clone:002-120-A09 Similar to eukaryotic translation initiation factor
					trans-regulation	Os12g0212100 Os11g0267300 Os06g0319600 Os04g0252200 Os06g0563300 Os08g0307400 Os05g0574550 Os04g0107900 Os06g0163400 Os08g0243100 Os04g0448900 Os06g0103300 Os02g0109100 Os03g0315800 Os09g0438400 Os10g0556600	NUDIX hydrolase domain Similar to Endonuclease III homologue Poly(A) polymerase Similar to CPSF160%3B nucleic acid binding Similar to serine/threonine protein phosphatase Phosphoinositide 3-kinase, accessory Hypothetical gene Non-protein-coding transcript Thioredoxin domain 2 containing protein 4′-phosphopantetheinyl transferase Similar to Zeaxanthin epoxidase Homogentisate 1,2-dioxygenase Similar to diphosphomevalonate decarboxylase Similar to 30S ribosomal protein S1 Choline/ethanolamine kinase CCR4-Not complex
4	Os01g0704250-00	43.22	10.33	Annotated lncRNA	cis-regulation	Os01g0702900 Os01g0703000 Os01g0704100 Os01g0703400 Os01g0706200	Similar to Sucrose-phosphate synthase SRP RNA 3′ adenylating enzyme Long-distance nitrate transport Farnesyl diphosphate synthetase N-terminal domain containing protein

## Supplementary Materials

The following supporting information can be downloaded at: https://www.mdpi.com/article/10.3390/plants11233223/s1, Figure S1: Expression validation of sequencing results using qPCR; Figure S2: Identification of LNC-037529 in different rice varieties; Figure S3: KEGG enrichment analysis of all DELs target genes; Figure S4: Schematic diagram of rice seed structure; Table S1: Statistical analysis of lncRNA sequencing data from Two samples with three biological replicates; Table S2: Significantly DELs during seed aging; Table S3: Cis- and trans-regulated target genes of DELs during seed aging; Table S4: Functional analysis of cis- and trans-regulated target genes of significantly up-regulated lncRNAs; Table S5: Go functional analysis of trans and cisregulated target genes of significantly downregulated lncRNAs; Table S6: KEGG functional annotation of trans-regulated target genes of significantly downregulated lncRNAs; Table S7: The predicted lncRNAs function as ceRNAs of miRNAs; Table S8: The target mRNAs of miRNAs; Table S9: The interacted pairs of lncRNAs, miRNAs, and target mRNAs in ceRNA network; Table S10: Annotation of mRNAs in the ceRNA network; Table S11: GO functional annotation of mRNA in ceRNA network; Table S12: KEGG functional annotation of mRNA in ceRNA network; Table S13: All primers used in this study.

## Abbreviations

lncRNA	long non-coding RNA
lincRNA	intergenic lncRNA
lncNAT	long non-coding natural antisense transcript
miRNA	microRNA
ceRNA	competing endogenous RNA
ssRNA-seq	strand-specific RNA sequencing
AAT	artificial aging treatment
DEL	differentially expressed lncRNA
DEG	differentially expressed mRNA
RACE	rapid-amplification of cDNA ends
LTR	long terminal repeated
S50	aged seeds (50% germination percentage)
S96	new seeds (96% germination percentage)
ORF	open reading frame
FPKM	fragments per kilobase of exons per million fragments mapped
PFAM	database of protein families
CPC	coding potential calculator
KEGG	Kyoto Encyclopedia of Genes and Genomes
PCC	pearson correlation coefficient
GO	Gene Ontology
NER	nucleotide excision repair
qPCR	Quantitative Real-time PCR
KOBAS	KEGG Orthology-Based Annotation System
ROS	reactive oxygen species
ABA	abscisic acid
GA	gibberellin
