# Supplementary figures and images for: Identification and Functional Analysis of Long Non-Coding RNA (lncRNA) in Response to Seed Aging in Rice
Source: Plants (Basel). 2022 Nov 24;11(23):3223. doi: 10.3390/plants11233223 (PMC9737669; doi:10.3390/plants11233223)

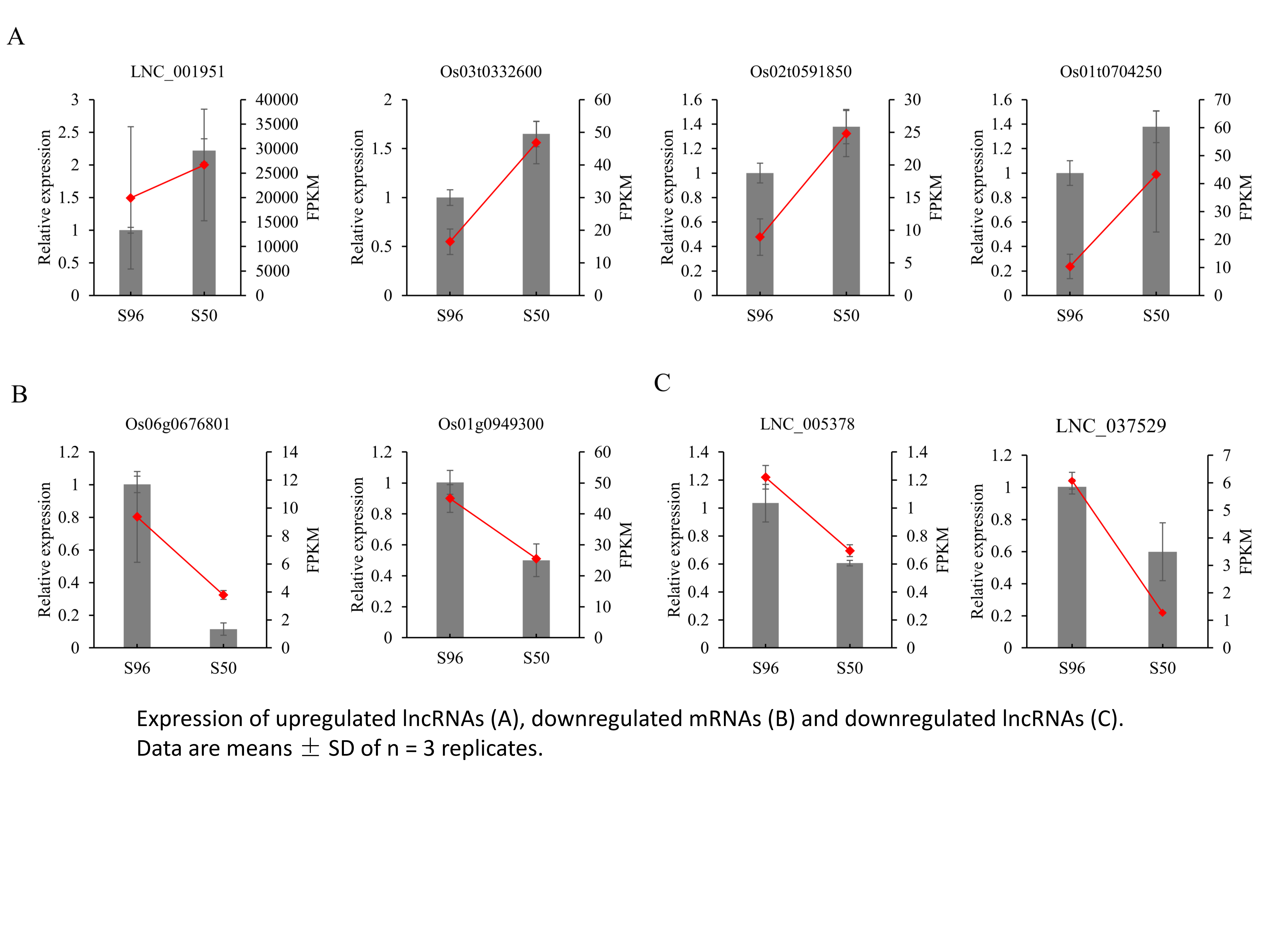

Supplement: Supplementary file 1 [file plants-11-03223-s001.zip › Supplementary Figure S1 Expression validation of sequencing results using qPCR. .png]

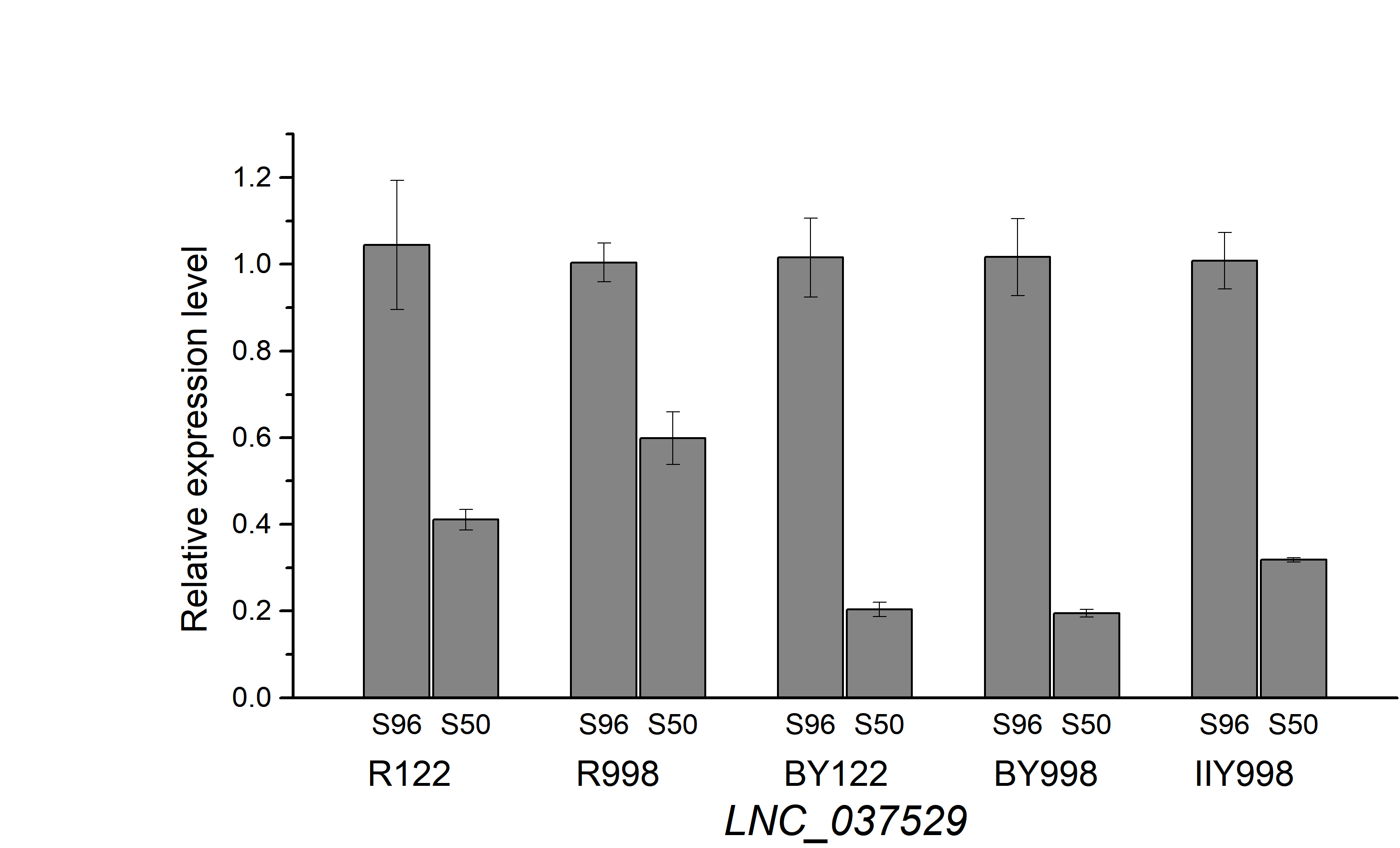

Supplement: Supplementary file 1 [file plants-11-03223-s001.zip › Supplementary Figure S2 Identification of LNC-037529 in different rice varieties .png]

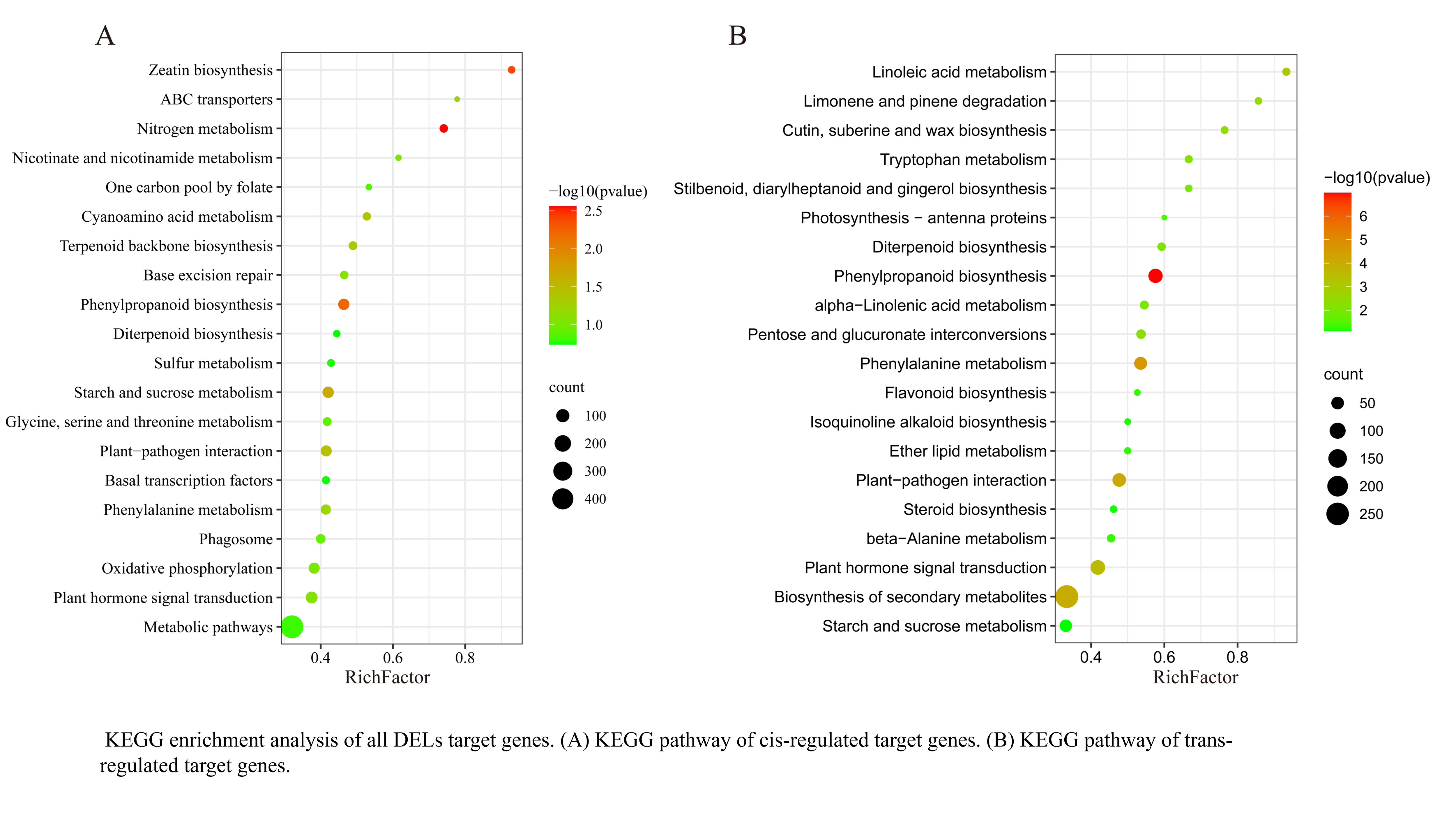

Supplement: Supplementary file 1 [file plants-11-03223-s001.zip › Supplementary Figure S3 KEGG enrichment analysis of all DELs target genes.png]

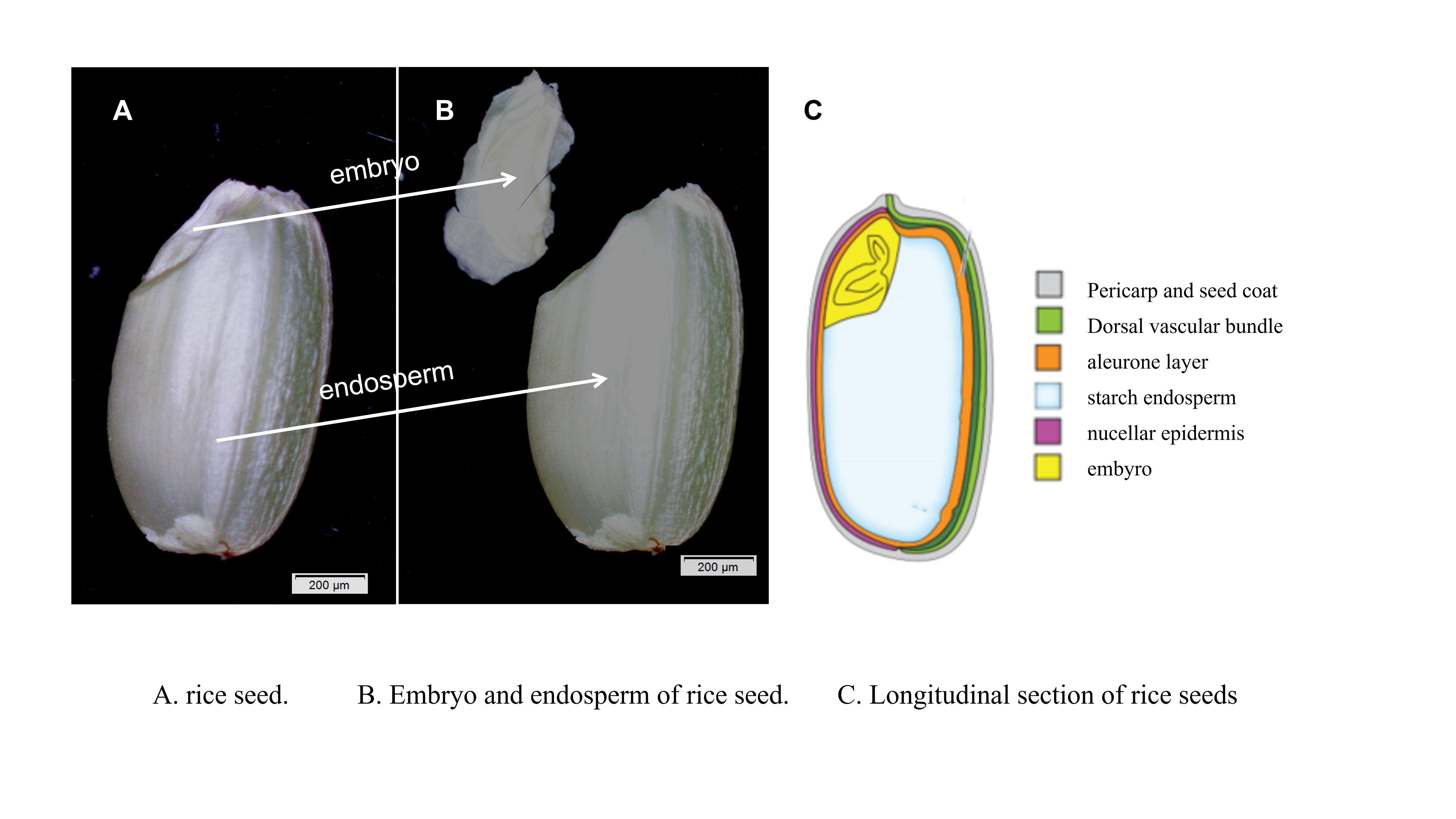

Supplement: Supplementary file 1 [file plants-11-03223-s001.zip › Supplementary Figure S4 Schematic diagram of rice seed structure.png]
